# Effects of Dietary Level of Corn Bran on Laying Performance and Cecum Microbial Communities in Laying Ducks

**DOI:** 10.3390/ani13233716

**Published:** 2023-11-30

**Authors:** Jia Hou, Qiufeng Zeng, Xuemei Ding, Shiping Bai, Jianping Wang, Huanwei Peng, Li Lv, Yue Xuan, Tao Zeng, Yong Tian, Lizhi Lu, Keying Zhang

**Affiliations:** 1 Key Laboratory for Animal Disease—Resistance Nutrition of China Ministry of Education, Animal Nutrition Institute, Sichuan Agricultural University, 211 Huimin Road, Wenjiang District, Chengdu 611130, China; houjia_kdq@126.com (J.H.); zqf@sicau.edu.cn (Q.Z.); dingxuemei0306@163.com (X.D.); shipingbai@sicau.edu.cn (S.B.); wangjianping1983@hotmail.com (J.W.); phw@sicau.edu.cn (H.P.); lvlisunny2012@163.com (L.L.); xuanyuede1007@hotmail.com (Y.X.); 2 Institute of Animal Husbandry and Veterinary Science, Zhejiang Academy of Agricultural Sciences, Hangzhou 310021, China; zengtao4009@126.com (T.Z.); tyong@zaas.ac.cn (Y.T.); lulizhibox2@163.com (L.L.)

**Keywords:** corn bran, laying duck, laying performance, microbial communities

## Abstract

**Simple Summary:**

Increasing numbers of livestock and aquaculture are resulting in insufficient feed. As a result, the use of unconventional raw materials is receiving increased attention. Corn bran (CB) is a by-product of corn starch production, which is high in yield and dietary fiber but low in price. Whether CB can be used in laying ducks’ diet and its maximum level remain unknown. In this study, the highest dietary CB level was set at 12%, which is double its maximum level in the laying duck industry. The results showed that the inclusion of 3%, 6%, 9%, and 12% CB in laying ducks’ diet under iso-energy and iso-nitrogen conditions had no adverse impacts on laying performance. Feeding ducks a 12% CB diet also improved digestive organ development, cecal beneficial bacterium population and beneficial metabolite production, albumen height, Haugh unit, and yolk color of eggs. The data suggested that the level of CB can reach up to 12% in a laying duck diet under iso-energy and iso-nitrogen conditions.

**Abstract:**

The application of corn bran (CB) to laying ducks via iso-energy and iso-nitrogen diets is rarely reported. Six hundred laying ducks (49 weeks) were equally assigned to five treatments: the control group with 0% CB and the other four groups with 3%, 6%, 9%, and 12% CB. The experiment lasted for 11 weeks. With the increase in CB, the relative weight of the proventriculus, gizzard, and ileum and the content and proportion of butyric acid in the cecal digesta were quadratically changed (*p* < 0.05), and the highest value was observed in the 12% CB group. Compared with the control, the 12% CB group showed decreased Deferribacteres, Spirochaetota, and Fusobacteriota at the phyla level and showed increased *Pediococcus* and decreased *Bifidobacterium* and *Rikenellaceae_RC9_gut_group* at the genus level (*p* < 0.10); the 12% CB group also showed 46 different metabolites, which are related to *Lactobacillus* and *Pediococcus* (*p* < 0.05). The 12% CB group showed increased (*p* < 0.05) albumen height at week 8 and yolk color at weeks 4 and 8 compared with the control. Overall, dietary inclusion of 3% to 12% CB is a possible feeding strategy for laying ducks under iso-energy and iso-nitrogen conditions, and the 12% CB group was more effective.

## 1. Introduction

Increasing numbers of livestock and aquaculture are resulting in insufficient feed. Therefore, the development and utilization of unconventional raw materials are important for the health and sustainability of animal husbandry.

Unconventional raw materials are usually by-products of the agricultural and food industries. Corn bran (CB) is a by-product of the corn starch obtained via wet milling [1], with a production of about 60 to 70 g per kg of corn [2]. In 2008, 25.6 × 10^6^ t of corn was processed through wet milling, producing about 0.341 × 10^6^ t of CB in America [3]. Globally, however, CB production is projected to be even larger. Traditional uses of CB include animal feed because of its low price. In truth, the amount of CB used in animal feed is limited because of its high fiber content, which has a negative impact on nutrient utilization and has been recognized as an anti-nutritional factor in the past few decades [4]. However, in recent years, fiber feedstuffs have received particular attention due to their functional value in improving digestive organ development, regulating gut health, promoting growth performance, and maintaining animal overall health in monogastric animals [5,6,7]. Currently, research into fiber feedstuffs in poultry mainly focuses on broilers [8] and meat ducks [9,10]. CB is an important source of fiber, including 10~20% cellulose and 30~40% hemicellulose, and mainly comprises arabinoxylan [11]. Arabinoxylan is good for animal health because it serves as a microbial fermentation substrate in the gut. Previous studies revealed that a post-weaning diet containing 5% CB altered intestinal microbial diversity [12,13], increased butyrate and total short-chain fatty acid (SCFA) production [13], and promoted an anti-inflammatory response to some extent in piglets [12]. To our knowledge, the application of CB as a fiber feedstuff in animal feed is lacking, especially in laying ducks. This leads to a lack of data to support CB’s use in the laying duck industry.

Considering the literature and the application potential of CB in the laying duck industry, the present study hypothesized that incremental dietary CB levels (0%, 3%, 6%, 9%, and 12%) would have no adverse effects on laying performance in ducks because of its beneficial impacts on intestinal organ development, intestinal microbial communities, and intestinal volatile fatty acid (VFA) profiles. In this study, we set a maximum CB level of 12%, which is double its maximum level in the laying duck industry. This study aimed to explore how CB level impacts production performance, digestive organ development, and the cecal microbial communities of laying ducks and finally find the optimal CB level for laying ducks.

## 2. Materials and Methods

### 2.1. Animals, Diets, and Management

All experimental procedures involved in this study were approved by the Institutional Animal Care and Use Committee of Sichuan Agricultural University (Chengdu, China; Ethic Approval Code: SICAUAC201910-1).

A total of 600 commercial laying ducks (Shendan-II, a new variety of laying ducks in China) at 49 weeks of age were randomly assigned to 5 dietary treatments based on their initial laying performance. Each treatment included 6 replicates, and each replicate had 10 cages (2 ducks/cage, 35 cm × 30 cm × 35 cm). The control group had 0% CB in the basal diet, and the other four groups included 3%, 6%, 9%, and 12% CB, respectively. The analyzed composition of corn bran used in this experiment is given in Table 1. The duration of this experiment was 11 weeks.

The diets were formulated to meet the nutrient requirements of laying ducks following the recommendation of Chinese laying ducks’ nutrient requirements (2021) [16]. The diet composition and nutrient levels are shown in Table 2. All diets were iso-nitrogen and iso-energy diets by switching the soybean oil with increased CB. All the diets were processed into pellets with a diameter of 4.5 mm. Ducks were reared in a controlled environment with a 16 h constant-light schedule (04:00–20:00) and free access to water and feed.

### 2.2. Production Performance of Laying Ducks

The number of eggs and ducks and egg weight were recorded daily, while the feed intake was recorded weekly for each replicate. The laying rate, egg weight, egg mass (egg weight per bird per day), average daily feed intake (ADFI), feed conversion ratio (FCR; g feed/g egg), mortality, and elimination rate were calculated.

### 2.3. Egg Quality

During the 4th, 8th, and 11th weeks, three eggs with an average weight in each replicate were selected for the egg quality analysis. Firstly, all eggs were examined to determine the shell quality characteristics, such as shell-breaking strength and shell thickness. Shell-breaking strength was measured using a shell force gauge model II (Robotmation Co., Ltd., Tokyo, Japan). The shell thickness was measured at the small end, equatorial region, and large end using a shell thickness gauge (Robotmation Co., Ltd., Tokyo, Japan). Secondly, the albumen height, Haugh unit, and yolk color were evaluated using an egg multi-tester (EMT-5200; Robotmation, Co., Ltd., Tokyo, Japan).

### 2.4. Relative Digestive Organ Weight and Length

After 11 weeks of rearing, 1 laying duck was randomly selected for each replicate, individually weighed, stunned, and exsanguinated (*n* = 6, 6 ducks per treatment). After dissection, the proventriculus, gizzard, small intestine, and cecum were instantly removed. After removing the contents, the weights of the proventriculus, gizzard, small intestine, and cecum and the length of intestinal segments were recorded. Based on the previous data, the relative weight (g/100 g body weight) and length (cm/100 g body weight) of intestinal segments, including the duodenum (from the gizzard to the bile duct), jejunum (from the bile duct to the Meckel’s diverticulum), ileum (from the Meckel’s diverticulum to the ileocecal junction), and cecum, were calculated.

### 2.5. Cecum Digesta Sample Collection and Measurement

The cecum digesta was collected, immediately frozen in liquid nitrogen, and stored at −80 °C until analysis. Subsequently, the cecum digesta samples were used for 16S rRNA, VFA, and metabolite analysis.

#### 2.5.1. Volatile Fatty Acid

VFA concentrations in cecal digesta were analyzed using the previously described method [17]. Approximately 0.6 g of sample was diluted in 1.5 mL of ultrapure water and homogenized via ultrasonic oscillation. All samples were stood for 30 min and then centrifuged at 20,000× *g* for 15 min. Then, 1 mL of supernatant was mixed with reagents (0.2 mL of 25% metaphosphoric acid and 23.3 μL of 210 mmol/L crotonic acid). After being mixed homogeneously and incubated at 4 °C for 30 min, the tube was centrifuged at 20,000× *g* for 10 min. Then, 0.3 mL of supernatant was collected and mixed with 0.9 mL of methanol, centrifuged at 10,000× *g* for 5 min, and filtered through a 0.22 μm filter. The supernatant was quantified using a gas chromatographic system (VARIAN CP-3800, San Diego, CA, USA). The VFA concentrations were expressed as μmol/g of digesta in the cecum.

#### 2.5.2. 16S-rRNA-Based Microbial Community Analysis

Cecal digesta samples were analyzed for the microbial community by Biotree Biomedical Technology Co., Ltd. (Shanghai, China) Total genomic DNA was extracted from cecal digesta using the TIANamp Soil DNA Kit (Tiangen BiotECH (Beijing) Co., Ltd., Beijing, China). Then, the V3-V4 variable regions of the bacterial 16S rRNA gene were amplified with universal primers (341F 5′-CCTACGGGNGGCWGCAG-3′ and 806R 5′-GGACTACHVGGGTWTCTAAT-3′) with barcodes unique to each sample. The amplicon sequencing with the Illumina HiSeq 2500 platform and bioinformatics were carried out. Quantitative Insights Into Microbial Ecology (QIIME, v1.9.1) was used for data analysis. Demultiplexing and quality filtering of the raw reads were performed with the FLASH, Trimmomatic, and UCHIME programs. The high-quality sequences were clustered into the operational taxonomic unit (OTU) using UCLUST at 97% similarity. OTU were then taxonomically classified using an RDP Classifier against a curated Green Genes database with a bootstrap cutoff of 80%. The weighted UniFrac distance metrics-based principal coordinate analysis (PCoA) plots showing microbial β-diversity were generated in EMPeror. Additionally, the analysis of microbial α- and β-diversity was performed with QIIME 2 (Version 1.7.0), and Tax4Fun predicted the microbial community functional profile.

#### 2.5.3. Cecal Digesta Metabolomics

The metabolomics analysis of cecal digesta was based on liquid chromatography mass spectrometry (LC-MS) and performed by Biotree Biomedical Technology Co., Ltd. (Shanghai, China). About 50 mg of digesta was mixed with 1000 μL of extract solution in an EP tube, homogenized at 35 Hz for 4 min, and sonicated for 5 min. The homogenization and sonication were repeated three times in an ice-water bath. Then, those samples were incubated at −40 °C for 1 h and subsequently centrifuged at 12,000 rpm for 15 min at 4 °C. The supernatant was used for analysis. In addition, quality control samples were also prepared for all samples. The UHPLC system (Vanquish, Thermo Fisher Scientific, Waltham, MA, USA) was used for LC-MS/MS analyses with a UPLC BEH Amide column (2.1 mm × 100 mm, 1.7 μm) coupled to a Q Exactive HFX mass spectrometer to acquire MS/MS spectra in an information-dependent acquisition mode. Finally, the data were processed using R 4.0.5 after the raw data were converted to the mzXML format using ProteoWizard.

### 2.6. Statistical Analysis

All data were analyzed using SAS statistical software (SAS 9.4, Inst. Inc., Cary, NC, USA). The experimental unit was the replicate (*n* = 6). The effect of treatment was analyzed using the MIXED procedure. Meanwhile, the analysis of linear and quadratic CB levels was conducted using orthogonal polynomial contrasts with the command CONTRAST. An independent two-sample *t*-test was used for microbial diversity analysis. The PROC GLIMMIX with gamma transformation was used when the residuals were nonnormal. *p* < 0.05 was considered significant; meanwhile, 0.05 ≤ *p* < 0.10 was considered a tendency. Additionally, function prediction was analyzed using a bootstrap Mann–Whitney U test based on gene distribution, with cutoffs of *p* < 0.01, false discovery rate < 0.1, and mean counts > 10.

## 3. Results

### 3.1. Laying Performance

The response of laying ducks to dietary CB levels is shown in Table 3. Overall data showed no significant effect of dietary treatments on laying performance in ducks except for ADFI. During weeks 5 to 8, the highest (*p* < 0.05) ADFI was observed in ducks fed on a diet with 6% and 12% CB compared to those in the 9% CB diet. During weeks 9 to 11, the significantly (*p* < 0.05) lowest ADFI was observed in ducks fed on a diet with 9% CB compared to other groups. Overall data showed higher (*p* < 0.05) ADFI in the 6% CB group than those of the 3% and 9% CB groups.

### 3.2. Egg Quality

The effects of CB levels on egg quality are shown in Table 4. The eggshell thickness was quadratically changed (*p* = 0.056; *p* < 0.05; *p* < 0.05) in weeks 4, 8, and 11. In the 4th week, the highest (*p* < 0.05) eggshell thickness was in the 6% CB group, and the lowest (*p* < 0.05) one was in the 12% CB group, which was lower (*p* < 0.05) than the 3% and 9% CB groups. In the 8th week, the eggshell thickness in the 0%, 6%, and 9% CB groups was higher (*p* < 0.05) than that in the 12% CB group. The eggshell breaking strength linearly decreased in week 11 (*p* = 0.086), where the eggshell breaking strength in the 0% CB group was higher (*p* < 0.05) than that in the 6% CB group. A linearly increased (*p* < 0.05) effect was observed on the albumen height and Haugh unit in week 8. For albumen height, the highest albumen height was observed in the 12% CB group in the 8th week, which was higher (*p* < 0.05) than the other groups except for the 9% CB group. For the Haugh unit, the 12% CB group was higher (*p* < 0.05) than the 3% CB group in the 8th week. A linearly increased (*p* < 0.05) effect on the yolk color was observed in the 4th week. Yolk color in the 0% and 3% CB groups was lower (*p* < 0.05) than that in the 12% CB group in week 4, while in the 8th and 11th weeks, the highest (*p* < 0.05) yolk color was observed in the 3% CB group.

### 3.3. Digestive Organs Index

As shown in Table 5, as the CB levels increased, the relative weight of the proventriculus, gizzard, and ileum showed a quadratic change (*p* < 0.05). The highest relative weights of the proventriculus and gizzard were in the 12% CB group, which were higher (*p* < 0.05) than other groups. Furthermore, both the 0% CB and 12% CB groups showed a higher (*p* < 0.05) relative weight of the ileum compared with other groups.

### 3.4. Volatile Fatty Acid Concentration in Cecal Digesta

As shown in Table 6, a quadratic change (*p* < 0.05) of total SCFA, acetic acid, propionic acid, and butyric acid concentrations; the proportion of butyric acid; and the branch-chain fatty acid (BCFA)-to-SCFA ratio were observed as the CB levels increased in diets. Ducks that were fed diets with 0% CB and 12% CB had higher (*p* < 0.05) total SCFA, acetic acid, and propionate concentrations than other groups. At the same time, the 12% CB group had the highest (*p* < 0.05) concentration and proportion of butyric acid compared with the other groups. Meanwhile, the valeric acid concentration was significantly enhanced (*p* < 0.05) in the 6% and 12% CB groups compared with the 3% CB group. Furthermore, ducks that were fed 3% CB had the highest BCFA-to-SCFA ratio, which was higher (*p* < 0.05) than other groups except for the 6% CB group.

### 3.5. Cecal Digesta Microbiota

**Figure 1:**
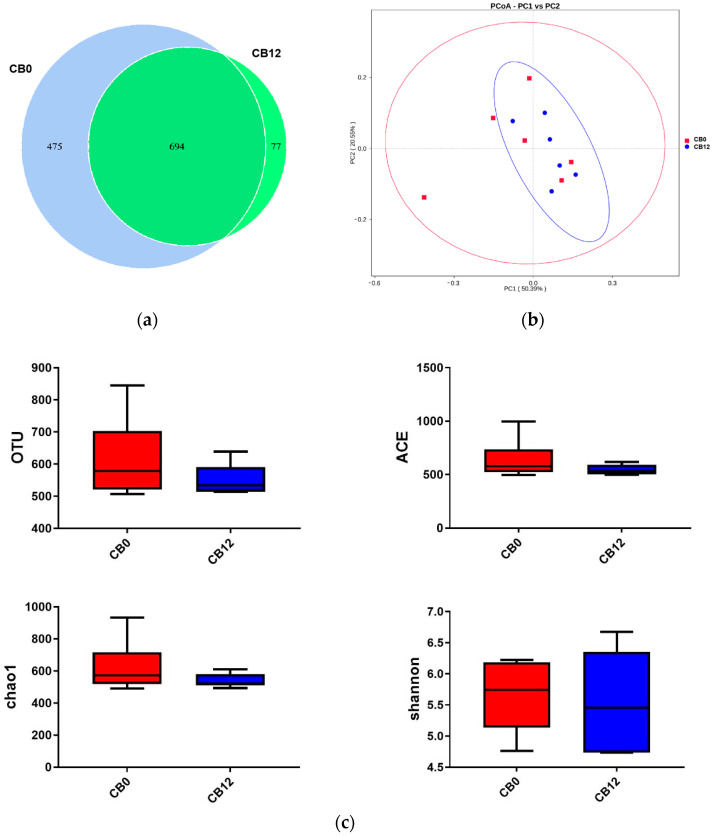
Venn diagram, PCoA, and average richness and diversity of the cecum digesta bacterial communities between groups. (**a**) Venn diagram between groups. (**b**) PCoA between groups. (**c**) average richness and diversity at the 3% dissimilarity level. CB0, corn bran is 0%; CB12, corn bran is 12%; PCoA, principal coordinate analysis; OTU, operational taxonomic unit; and ACE, abundance-based coverage estimator.

After quality control, 760,958 high-quality sequences were obtained, averaging 63,413 sequences per sample. The Venn diagram (Figure 1a) indicated that 1169 and 771 OTUs were observed from ducks that were fed diets with dietary CF levels of 0% CB and 12%, respectively, of which 694 OTUs were shared between groups. Meanwhile, the PCoA (Figure 1b) demonstrated an extensive bacterial community in the 0% CB group. However, there was no significant difference (*p* > 0.05, Figure 1c) in the OTU number, abundance-based coverage estimator (ACE), Chao 1, and Shannon between groups, although the OTU, ACE, and Chao 1 were numerically higher in the 0% CB group than those in the 12% CB group.

**Figure 2:**
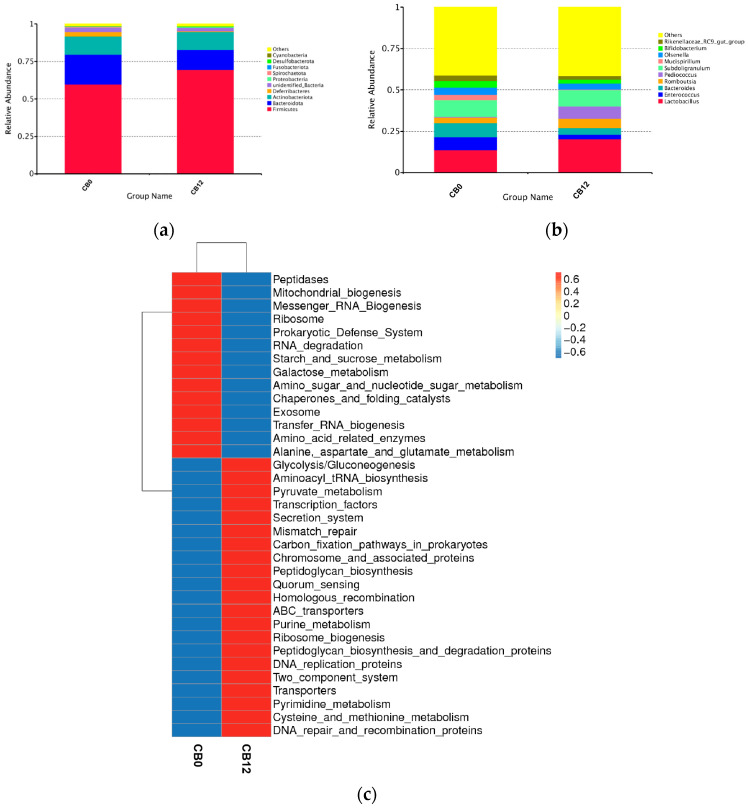
The top 10 phyla and genera and their function prediction in cecum digesta between groups. (**a**) Top 10 phyla between groups. (**b**) Top 10 genera between groups. (**c**) Function prediction. Log 10 transform was used for each functional gene. CB0, corn bran is 0%; CB12, corn bran is 12%.

The microbial composition showed that Firmicutes, Bacteroidota, and Actinobacteriota were the three dominant phyla, with a relative proportion of more than 90%, in laying ducks (Table 7, Figure 2a). The relative abundance of Deferribacteres, Spirochaetota, and Fusobacteriota was higher (*p* < 0.05) in the 0% CB group than in the 12% CB group. At the genus level (Table 8, Figure 2b), a higher abundance of (*p* < 0.05) *Pediococcus* was found in the 12% CB group, while the 0% CB group tended to increase the abundance of *Bifidobacterium* and *Rikenellaceae_RC9_gut_group* (*p* = 0.082; *p* = 0.085). Interestingly, although there was no statistical difference, ducks that were fed the diet with 0% CB numerically decreased the relative abundance of *Lactobacillus* (*p* = 0.339; reduced by 6.53%) but increased the relative abundance of *Bacteroides* (*p* = 0.113; increased by 4.44%) than that of the 12% CB group.

In addition, the function prediction results in Figure 2c showed distinct microbial functions between groups. The results suggested that bacteria in the 0% CB group were involved in metabolizing peptidases, starch, sucrose, and amino acids. In contrast, bacteria in the 12% CB group were involved in glycolysis, pyruvate metabolism, and quorum sensing.

### 3.6. Cecal Digesta Metabolomics and Metabolic Pathway

**Figure 3:**
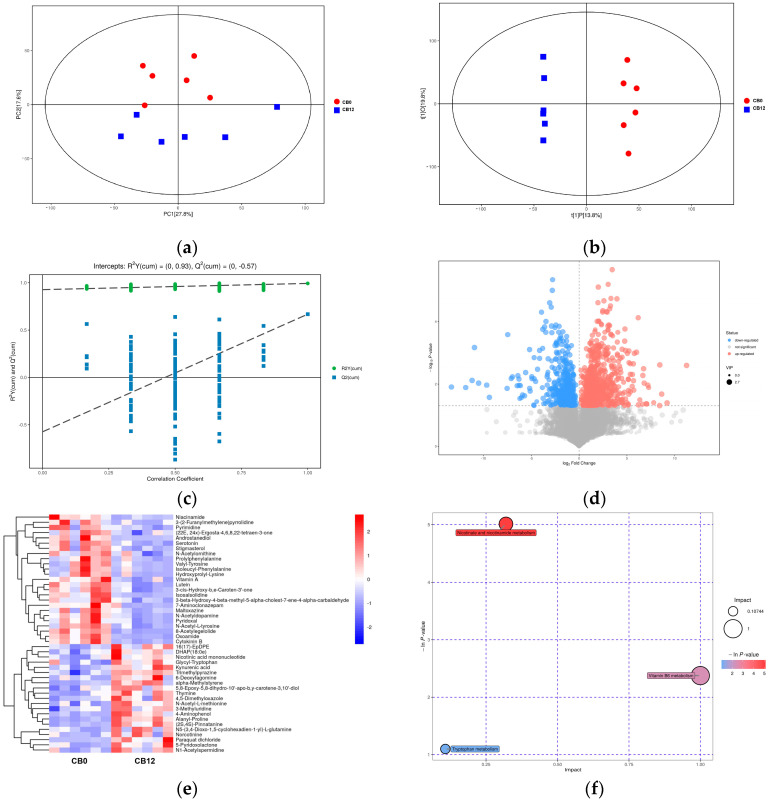
The metabolites and their metabolic pathway analysis in cecal digesta. (**a**) Score scatter plot of PCA model. (**b**) Score scatter plot of OPLS-DA model. (**c**) Permutation test of OPLS-DA model. (**d**) Volcano plots. The red dots mean up-regulated, and the blue dots mean down-regulated. (**e**) Hierarchical clustering analysis. (**f**) Topology analysis of metabolic pathway. The *X*-axis and *Y*-axis, respectively, represent the rich factor and the negative nature logarithm of the *p*-value. PCA, principal component analysis; OPLS-DA, orthogonal projections to latent structures–discriminant analysis.

As shown in Figure 3, cecal digesta samples were analyzed in positive ionization mode, and principal component analysis (PCA) and orthogonal projections to latent structure–discriminant analysis (OPLS-DA) were used to visualize the LC-MS dataset. The PCA score plots (Figure 3a) indicated distinct metabolites among treatments, and the supervised OPLS-DA score plots (Figure 3b) also showed separation among different groups. A permutation test of the OPLS-DA model among groups was conducted (Figure 3c). The results showed that the R2Y was close to 1, and the intercept of the Q2 and the *Y*-axis was negative, indicating no overfitting. The metabolites were displayed in the volcano plot (Figure 3d). Under the selection criteria of a *p*-value < 0.05, the number of differentially expressed metabolites between 0% CB and 12% CB was 46 (Figure 3e).

The pathway analysis based on the Kyoto Encyclopedia of Genes and Genomes pathway database showed multiple biochemical pathways between groups. Ducks in the 0% CB and 12% CB groups differed in nicotinate and nicotinamide metabolism, vitamin B6 metabolism, and tryptophan metabolism (Figure 3f), whereas the 12% CB group had lower nicotinic acid mononucleotide but higher niacinamide, pyridoxal, and serotonin as compared with the 0% CB group.

### 3.7. Pearson Correlation between Cecal Digesta Metabolomics and Microbes

**Figure 4:**
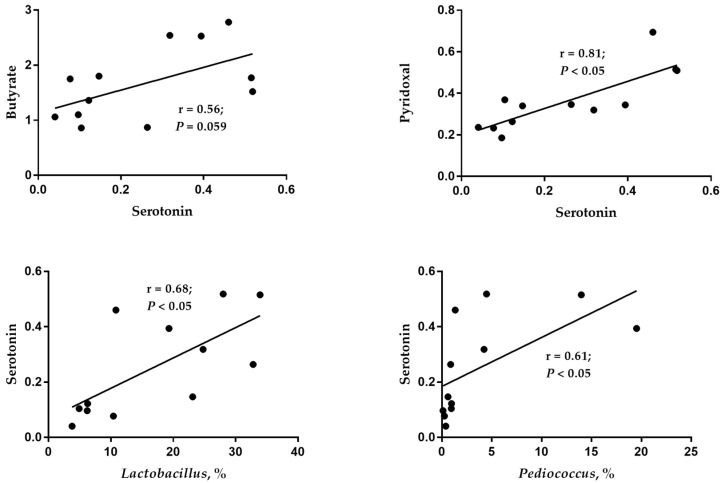
Pearson correlation between different metabolites and microbial profiles in cecal digesta of laying ducks.

As shown in Figure 4, butyric acid is positively related to serotonin (r = 0.56, *p* = 0.059). At the same time, serotonin positively correlated with *Lactobacillus* (r = 0.68; *p* < 0.05), *Pediococcusr* (r = 0.61; *p* < 0.05), and pyridoxal (r = 0.81; *p* < 0.05).

## 4. Discussion

This study evaluated the effects of dietary CB levels on laying performance, organ development, cecum microbial communities, VFA profiles in cecal digesta, and egg quality in laying ducks. To our knowledge, this study is the first trial to explore whether CB can be used in laying ducks’ diet and its maximum level, which will guide the use of CB in the laying duck industry.

Corn bran is an insoluble fiber source. A previous study indicated that based on a typical corn–soybean meal diet for laying ducks, the substitution of soybean meal and wheat bran by different levels of the distiller dried grains with solubles (6%, 12%, 18%, 24%, and 30%) under iso-energy and iso-nitrogen diets showed no effect on laying rate [18]. However, an energy- and nutrient-reduced diet containing 10% lignocellulose in laying hens displayed a higher laying rate than the control group in 23–52 weeks [19]. In the present study, there were no significant differences in indexes relative to laying performance except for ADFI among groups. However, the 12% CB group numerically increased duck-housed egg production by 2.46% and duck-housed egg number by two eggs compared with the control (0% CB) group, suggesting that the inclusion of 12% CB in the diet had a positive impact on the laying performance. Usually, egg production is driven by dietary energy and nutrient ingestion. The results showed that there were no differences in ADFI between the 0% CB and 12% CB groups in each stage, which suggested that feed intake was not the only factor that drove laying performance when ducks consumed a diet that included high crude fiber by adding 12% CB. In this study, the incremental CB level (from 0% to 3%, 6%, 9%, and 12%) gradually increased feed cost (2.57, 2.58, 2.60, 2.61, and 2.63 RMB/kg), displaying an increased net profit of RMB −2.31, −0.73, −0.94, and 1.31 in the 3%, 6%, 9%, and 12% CB groups, respectively, which indicated that only the 12% CB group had a positive effect on economic benefit. In conclusion, the 12% CB group displayed a beneficial effect rather than an anti-nutrition effect on laying performance in laying ducks.

Corn bran is a fiber source for animal feed. The CB used in the current study contained 12.4% crude fiber. However, the effect of CB on egg quality in laying ducks is rarely reported. In the current study, as the dietary CB level increased, the eggshell thickness quadratically changed in weeks 4, 8, and 11. The 12% CB group illustrated the lowest eggshell thickness on week 8, which was lower than the control (0% CB) group (0.34 vs. 0.38 mm). This result was consistent with the previous studies that were conducted with other fiber sources in laying hens, in which adding incremental sugar beet pulp (0%, 5%, 10%, 15%, and 20%) to the laying hen diet during 24 to 27 weeks of age linearly decreased the shell thickness [20]. However, the slightly decreased shell thickness in the 12% CB group had no influence on shell-breaking strength compared to the control group in week 8, despite thinner shells always being accompanied by lower shell-breaking strength in theory [21]. Additionally, the decreased shell thickness (0.37 vs. 0.42 mm) in the 6% CB group led to a decreased tendency of shell-breaking strength compared with the control (0% CB) group in week 11. The results of the 0% CB and 6% CB groups agreed with a previous study about fiber source feedstuffs, in which a laying hen diet that contained 5% sunflower hull decreased shell thickness compared with the control [22]. However, the mechanism behind the dosage-dependent effect of CB on shell thickness and shell-breaking strength is still unknown. As is known, the higher the albumen height and Haugh unit, the better the egg quality. The 12% CB group had the highest albumen height and Haugh unit in week 8, indicating better egg quality in this group. In this study, the yolk color was improved as the CB level increased in week 4, and the yolk color was greatly enhanced in the 12% CB group compared with the control (0% CB) group in weeks 4 and 8. These observations were expected because CB contains relatively high xanthophyll content, which is an important contributor to yolk pigmentation [23]. In conclusion, the inclusion of 12% CB in a duck diet showed positive effects on albumen height, Haugh unite, and yolk color and had no adverse effects on shell-breaking strength compared with the 0% CB group.

The proventriculus and gizzard are the characteristic components of the digestive tract in poultry [8]. As is known, the proventriculus, gizzard, and intestine play an important role in the digestion and absorption of nutrients and are, thus, the organs most influenced by dietary changes [24]. Fiber type and feed particle size are the determining factors that stimulate the gizzard’s muscular activity, resulting in the altered size of those organs [25]. In the present study, the relative weights of the proventriculus and gizzard linearly increased as the CF level increased, and the highest relative weight was found in the 12% CB group, which was significantly higher than other groups. These results were consistent with previous studies about other fiber sources, in which the inclusion of 6% wood shavings, 3% oat hulls, or 3% soy hulls in the diet led to an increased size of proventriculus and gizzard in broilers as a logical result of an increased volume due to the slower passage rate of the almost-intact feed particles [26,27]. Additionally, the fiber source also impacts intestinal development in poultry, although the results are not always consistent. The inclusion of 3% oat hull in a broiler diet led to a lower relative length of the small intestine and a higher relative weight of the cecum; meanwhile, the inclusion of 3% soy hull had no impact on intestine development [27]. Chickens fed 3% wheat bran increased the relative weight of the small intestine [28]. In this study, there was no difference in the relative weight and length of the intestinal segments between the 12% and 0% CB groups. Still, the relative weight of the ileum was lower in the remaining groups than in the 0% CB and 12% CB groups, illustrating a fiber-level-dependent effect. The results suggested that the inclusion of 12% CB in the laying duck diet improved the development of digestive organs.

Dietary fiber, as the primary substance used for bacterial fermentation in the intestine, was reported to support the growth and establishment of beneficial microbes by providing extra fuel for their metabolism [6], which ultimately enhanced the production of SCFA [29,30,31,32]. In this study, the CB raw material contained 40.46%, 5.16%, and 35.30% of total dietary fiber, soluble dietary fiber, and insoluble dietary fiber, respectively. As a result, the total SCFA, acetic acid, propionic acid, and butyric acid concentrations and the BCFA-to-SCFA ratio quadratically changed as the CB level increased. The 0% CB and 12% CB groups had higher contents of total SCFA, acetic acid, and propionic acid than other groups, reflecting a better fermentation effect in these two groups. Notably, the 12% CB group also showed a higher butyric acid concentration and proportion than the 0% CB group, indicating a CB level dependence in VFA production in the cecum. A previous study indicated that SCFA can provide up to 5% to 15% of daily metabolizable energy for the maintenance energy requirements of birds [33]. Meanwhile, butyrate has the functions of anti-inflammatory activity, immune response modulation, and growth performance enhancement [34]. These functions of SCFA and butyrate partly explained how the 12% CB group maintained or numerically improved the laying performance compared with the control group.

The above results showed that the 12% CB group demonstrated beneficial effects on digestive organ development and cecal VFA production without adverse effects on laying performance. To further explore the mechanism behind this phenomenon, we further explored the cecal microbial communities and metabolites in the 0% CB and 12% CB groups. Intestinal microbial communities result from their adaptation to the environment and nutrients [35], showing a crucial role in manipulating host health and growth performance by regulating metabolism [36,37]. In this study, the PCoA showed an extensive range in the 0% CB group, although there was no difference in α-diversity, illustrating a distinct change in microbial composition between the 0% CB and 12% CB groups. In the present study, the three dominant phyla were Firmicutes, Bacteroidetes, and Actinobacteriota, which is consistent with the previous report on Shaoxing laying ducks [38]. Higher levels of Firmicutes but lower levels of Bacteroidetes were found when the ducks consumed a diet containing 12% CB, which was in line with the altered cecum microbial communities with incremental fiber in geese [12]. Deferribacteres, a harmful taxon [39], was proven to have a positive correlation with IL-1β expression [40]. In this study, ducks that were fed a diet including 12% CB resulted in lower Deferribacteres. Additionally, Spriochaetota and Fusobacteriota, phyla containing many pathogenic microorganisms [41,42], declined when ducks were fed a diet including 12% CB. These observations suggest the presence of better gut health when the ducks were fed the diet of the 12% CB group compared with that of the control (0% CB) group.

At the genus level, *Lactobacillus* was the dominant genus, illustrating 6.5% higher relative abundance in the 12% CB group than in the 0% CB group. Likewise, the genus of *Pediococcus* was also higher in the 12% CB group (7.42% vs. 0.54%). As is known, both *Lactobacillus* and *Pediococcus* are lactic acid bacteria with the function of producing lactate [43,44,45], which is beneficial for gut health by inhibiting the growth of putrefying bacteria [17,46]. Meanwhile, both *Lactobacillus* and *Pediococcus* have the function of improving intestinal VFA production [33,47]. The higher relative abundance of *Lactobacillus* and *Pediococcus* in the 12% CB group explained its higher cecal butyric acid production compared to the 0% CB group. Furthermore, *Bacteroides*, positively related to pro-inflammatory cytokines [48], exhibited a declining trend as the crude fiber increased. However, *Bifidobacterium*, which was set as a probiotic [49], was higher in the 0% CB group. As a whole, the 12% CB group had a higher level of beneficial bacteria. Interestingly, we observed an increased level of the *Rikenellaceae_RC9_gut_group* in the 0% CB group. Previous studies reported that the *Rikenellaceae_RC9_gut_group* widely participated in the metabolism of bile acid, protein, fat, and carbohydrates [50], and propionate, acetate, and succinate were the fermentation end products of this process [51]. It partly explained the higher predictive function of the metabolism of peptidases, starch, sucrose, and amino acids in the 0% CB group. In addition, the distinct predictive function between those two groups indicated that the 12% CB group had a robust predictive function of glycolysis and pyruvate metabolism related to fiber fermentation in the gut [52], which was reported to result in increased acetate, propionate, and butyrate concentrations [53] and subsequently benefit the health of the host. Notably, the 12% CB group displayed enhanced quorum sensing. This density-dependent cell-to-cell signaling occurs when the bacterial population reaches a threshold density [54]. The same phenomenon was observed in piglets due to the enhanced gut *Lactobacillus* [17], providing evidence for a link between higher *Lactobacillus* and the robust quorum sensing in the 12% CB group.

The cecal digesta metabolomics results revealed changes in cecal metabolites between the 0% CB and the 12% CB groups. Niacin is a vitamin B_3_ compound with anti-inflammatory and antioxidant effects [47]. Pyridoxal is a coenzyme of more than 260 enzymes and has antioxidant effects [55]. Therefore, the increased concentrations of niacinamide and pyridoxal in the 12% CB group indicated that the diet in this group was more beneficial for laying ducks’ health, which lays a foundation for its positive impact rather than an anti-nutrition effect on laying performance. Previous studies have shown that increasing dietary fiber content can enhance serotonin production by affecting microbial metabolites, such as increasing butyric acid content [56,57]. Likewise, when the crude fiber level increased from 2.84% to 3.94% by adding 12% CB, the serotonin content in cecal digesta increased in this study, and the serotonin content showed a positive correlation with the abundance of *Lactobacillus* and *Pediococcus* and the content of butyric acid in cecal digesta. In addition, as pyridoxal is a coenzyme in the process of serotonin synthesis, an increase in its content might be more conducive to the synthesis of serotonin. As a result, the current study observed a significant positive correlation between pyridoxal and serotonin. In conclusion, there were different impacts on the intestinal microbial composition and microbial derivative metabolites between the 0% CB and 12% CB groups, suggesting a beneficial effect on the intestinal health of ducks in the 12% CB group.

## 5. Conclusions

Collectively, increasing dietary CB inclusion from 0% to 12% is a possible feeding strategy under iso-energy and iso-nitrogen conditions, as the practice did not compress the laying performance of laying ducks. Feeding ducks a 12% CB diet also improved digestive organ development, cecal beneficial bacterium population and beneficial metabolite production, albumen height, Haugh unit, and yolk color of eggs. However, the maximum level of CB was 12% in the current study. Whether the dietary CB level can be higher than 12% needs to be studied further. Based on the findings in this study, it can be concluded that the CB level in a laying duck diet can reach up to 12% under iso-energy and iso-nitrogen conditions.

## Figures and Tables

**Table 1:** The analyzed composition of corn bran (%, air dry basis).

Items	Corn Bran ^1^
Composition	
Moisture	10.30
Gross energy, MJ/kg	15.69
Crude protein	9.76
Crude fat	3.94
Crude fiber	12.4
Neutral detergent fiber	41.47
Acid detergent fiber	13.40
Total dietary fiber	40.46
Soluble dietary fiber	5.16
Insoluble dietary fiber	35.30
Lysine	0.26
Methionine	0.17
Methionine + cystine	0.29
Mycotoxins ^2^, μg/kg	
Aflatoxin B_1_	ND (50)
Deoxynivalenol	ND (5000)
Zearalenone	298.4 (1500)

^1^ Corn bran was purchased from Shendan Health Food Co., Ltd. (Xiaogan, China). The contents of crude fiber, total dietary fiber, soluble dietary fiber, and insoluble dietary fiber were measured at the Guangxi Center for Analysis and Test Research, and other nutrient compositions were measured at the Beijing Institute of Animal Sciences (Chinese Academy of Agricultural Sciences), according to AOAC (2007) [14]. ^2^ Mycotoxin content in CB was detected using High-Performance Liquid Chromatography and mass spectrometry methods at Hangzhou ZhongkeYoulong Ltd. (Hangzhou, China) The contents in brackets are the limited values of different mycotoxins according to hygienic standards for feeds (National Standard GB 13078-2017 [15]).

**Table 2:** Composition and nutrient levels of the experimental diets (as fed basis).

Items	Corn Bran Level, %				
	0	3	6	9	12
Ingredients, %					
Corn (7.8% crude protein)	40.71	37.03	33.36	29.68	26.00
Soybean meal (43% crude protein)	23.00	23.00	23.00	23.00	23.00
Wheat flour	15.00	15.00	15.00	15.00	15.00
Rice bran	2.00	2.00	2.00	2.00	2.00
Sunflower meal	4.00	4.00	4.00	4.00	4.00
Corn gluten meal	1.50	1.50	1.50	1.50	1.50
Corn bran	0.00	3.00	6.00	9.00	12.00
Soybean oil	2.00	2.75	3.50	4.25	5.00
L-Lysine sulfate (70%)	0.25	0.25	0.26	0.26	0.26
DL-Methionine (99%)	0.21	0.21	0.21	0.21	0.21
Dicalcium phosphate	0.55	0.58	0.60	0.63	0.65
Limestone	8.58	8.59	8.59	8.60	8.60
Sodium chloride	0.40	0.40	0.40	0.40	0.40
Bentonite	0.80	0.70	0.59	0.49	0.38
Premix ^1^	1.00	1.00	1.00	1.00	1.00
Total	100.00	100.00	100.00	100.00	100.00
Nutrient level ^2^, %					
Dry matter	87.46	87.60	87.75	87.90	88.05
Metabolizable energy, MJ/kg	11.61	11.61	11.61	11.61	11.61
Crude protein	18.03	18.04	18.06	18.07	18.09
Calcium	3.50	3.50	3.50	3.50	3.50
Total phosphorus	0.50	0.50	0.50	0.50	0.50
Non-phytate phosphorus	0.23	0.23	0.23	0.23	0.23
True digestible lysine	0.85	0.85	0.85	0.85	0.85
True digestible methionine	0.44	0.44	0.44	0.44	0.44
True digestible methionine + cystine	0.69	0.68	0.68	0.67	0.67
True digestible threonine	0.53	0.53	0.53	0.53	0.53
True digestible tryptophane	0.17	0.17	0.17	0.17	0.17
Crude fiber	2.84	3.12	3.39	3.67	3.94
Neutral detergent fiber	9.52	10.58	11.64	12.70	13.76
Acid detergent fiber	4.73	5.00	5.26	5.53	5.79
Total dietary fiber	12.85	13.85	14.86	15.86	16.87
Soluble dietary fiber	2.10	2.07	2.04	2.02	1.99
Insoluble dietary fiber	10.74	11.77	12.80	13.83	14.86

^1^ Premix provided per kilogram diet: vitamin A, 9900 IU (retinyl acetate); vitamin D_3_, 4000 IU; vitamin E (α-tocopherol acetate), 40.0 IU; vitamin K_3_, 2.50 mg; vitamin B_1_, 2.00 mg; vitamin B_2_, 8.00 mg; nicotinic acid, 35.0 mg; vitamin B_6_, 3.00 mg; vitamin B_12_, 20.0 µg; biotin, 200 µg; calcium pantothenic acid, 20.00 mg; folic acid, 1.00 mg; zinc (zinc sulfate), 110 mg; iron (iron carbonate), 50.0 mg; manganese (manganese sulfate), 120 mg; copper (copper sulfate pentahydrate), 12.0 mg; iodate (potassium iodate), 1.20 mg; selenium (sodium selenite), 0.30 mg; carophyll red, 60 mg; carophyll yellow, 30 mg; and phytase, 2000 IU. ^2^ The nutrient levels were calculated values.

**Table 3:** Effects of dietary fiber on laying performance in laying ducks.

Items	Corn Bran Level, %					SEM	*p*-Value		
	0	3	6	9	12		ANOVA	Linear	Quadratic
Duck-housed egg production, %									
1–11 w	76.21	73.02	76.66	75.21	78.67	2.090	0.437	0.293	0.303
Duck-day egg production, %									
1–11 w	79.02	74.73	79.61	76.22	79.53	1.762	0.209	0.655	0.304
Number of duck-housed eggs, number/bird									
1–11 w	58.68	56.23	59.03	57.91	60.58	1.609	0.437	0.293	0.302
Egg weight, g									
1–11 w	71.75	72.49	71.75	72.45	71.95	0.513	0.732	0.832	0.591
Egg mass, g/d									
1–11 w	56.73	54.18	57.13	55.22	57.24	1.444	0.498	0.658	0.436
ADFI, g/d									
1–4 w	153.5	152.0	158.3	152.8	153.8	1.84	0.164	0.798	0.332
5–8 w	160.9 ^ab^	159.0 ^ab^	162.4 ^a^	157.3 ^b^	162.6 ^a^	1.30	0.036	0.798	0.333
9–11 w	160.8 ^a^	161.6 ^a^	165.2 ^a^	155.5 ^b^	160.6 ^a^	1.68	0.009	0.710	0.245
1–11 w	158.1 ^ab^	157.1 ^b^	161.6 ^a^	155.1 ^b^	158.8 ^ab^	1.38	0.040	0.910	0.758
FCR, g feed/g egg									
1–11 w	2.80	2.91	2.84	2.82	2.78	0.069	0.696	0.556	0.342
Mortality and elimination rate, %									
1–11 w	7.50	5.83	6.67	5.00	4.17	1.848	0.730	0.211	0.905

ADFI, average daily feed intake; FCR, feed conversion ratio. ^a,b^ Values within a row with no common superscripts differ significantly (*p* < 0.05).

**Table 4:** Effects of dietary fiber on egg quality in laying ducks.

Items	Corn Bran Level, %					SEM	*p*-Value		
	0	3	6	9	12		ANOVA	Linear	Quadratic
Shell thickness, mm									
4 w	0.39 ^abc^	0.40 ^ab^	0.41 ^a^	0.38 ^bc^	0.37 ^c^	0.010	0.035	0.041	0.056
8 w	0.38 ^a^	0.37 ^ab^	0.39 ^a^	0.40 ^a^	0.34 ^b^	0.014	0.039	0.173	0.039
11 w	0.42 ^a^	0.32 ^c^	0.37 ^b^	0.38 ^ab^	0.39 ^ab^	0.014	0.002	0.839	0.004
Shell-breaking strength, kg/cm^2^									
4 w	3.91	4.09	4.34	3.99	3.57	0.157	0.101	0.139	0.019
8 w	4.24	4.27	4.26	3.80	4.34	0.238	0.530	0.710	0.528
11 w	4.64	4.03	3.67	3.77	4.07	0.250	0.086	0.089	0.022
Albumen height, mm									
4 w	7.95	7.63	6.66	6.96	6.71	0.446	0.187	0.036	0.385
8 w	6.05 ^b^	5.88 ^b^	6.35 ^b^	6.60 ^ab^	7.14 ^a^	0.257	0.016	0.002	0.217
11 w	6.76	5.69	6.43	6.43	6.01	0.413	0.418	0.570	0.728
Haugh unit, Hu									
4 w	84.48	83.86	77.08	79.11	77.63	2.795	0.215	0.052	0.456
8 w	72.26 ^ab^	70.51 ^b^	74.96 ^ab^	77.23 ^ab^	80.32 ^a^	1.938	0.012	0.002	0.330
11 w	77.56	68.50	74.55	75.19	70.15	3.233	0.281	0.434	0.830
Yolk color									
4 w	12.17 ^b^	12.25 ^b^	13.06 ^a^	12.89 ^a^	13.11 ^a^	0.137	<0.01	<0.001	0.227
8 w	12.33 ^c^	14.11 ^a^	12.72 ^bc^	12.56 ^bc^	12.83 ^b^	0.170	<0.01	0.700	0.330
11 w	12.39 ^b^	14.00 ^a^	12.56 ^b^	12.50 ^b^	13.00 ^b^	0.224	<0.01	0.700	0.330

^a–c^ Values within a row with no common superscripts differ significantly (*p* < 0.05).

**Table 5:** Effects of dietary fiber on digestive organ development in laying ducks.

Items ^1^	Corn Bran Level, %					SEM	*p*-Value		
	0	3	6	9	12		ANOVA	Linear	Quadratic
Relative weight, g/100 g body weight									
Proventriculus	0.37 ^b^	0.36 ^b^	0.37 ^b^	0.34 ^b^	0.43 ^a^	0.020	0.042	0.139	0.035
Gizzard	1.87 ^b^	1.80 ^b^	1.85 ^b^	1.66 ^b^	2.35 ^a^	0.149	0.036	0.095	0.030
Duodenum	0.62	0.51	0.60	0.55	0.53	0.043	0.357	0.362	0.817
Jejunum	1.33	1.14	1.27	1.21	1.32	0.098	0.594	0.848	0.264
Ileum	1.37 ^a^	0.82 ^b^	0.97 ^b^	0.86 ^b^	1.40 ^a^	0.106	0.001	0.756	<0.001
Cecum	0.48	0.37	0.40	0.35	0.42	0.050	0.379	0.382	0.170
Relative intestinal length, cm/100 g body weight									
Duodenum	1.86	1.74	1.68	1.68	1.74	0.083	0.536	0.255	0.187
Jejunum	4.55	4.29	4.29	4.14	3.95	0.334	0.750	0.193	0.999
Ileum	4.11	3.95	3.89	3.64	4.04	0.179	0.325	0.391	0.149
Cecum	1.08	0.93	0.99	0.95	1.21	0.106	0.878	0.734	0.434

^1^ Based on the live body weight. ^a,b^ Values within a row with no common superscripts differ significantly (*p* < 0.05).

**Table 6:** Effects of dietary fiber on volatile fatty acids in cecal digesta in laying ducks (μmol/g).

Items	Corn Bran Level, %					SEM	*p*-Value		
	0	3	6	9	12		ANOVA	Linear	Quadratic
Total SCFA ^1^	147.35 ^a^	29.91 ^c^	60.50 ^b^	50.55 ^bc^	166.41 ^a^	9.702	<0.001	0.067	<0.001
Acetic acid	99.94 ^a^	20.93 ^c^	40.11 ^b^	35.14 ^bc^	111.55 ^a^	6.073	<0.001	0.063	<0.001
Propionic acid	32.36 ^a^	6.96 ^b^	15.25 ^b^	11.42 ^b^	32.14 ^a^	3.335	<0.001	0.707	<0.001
Butyric acid	15.04 ^b^	2.03 ^c^	5.15 ^c^	4.00 ^c^	22.73 ^a^	1.842	<0.001	0.006	<0.001
Valeric acid	0.98 ^abc^	0.57 ^c^	1.16 ^ab^	0.78 ^bc^	1.41 ^a^	0.183	0.033	0.078	0.124
Total BCFA ^2^	1.42	1.11	1.50	1.49	2.11	0.323	0.311	0.096	0.242
Isobutyric acid	0.55	0.44	0.66	0.64	0.70	0.119	0.538	0.189	0.839
Isovaleric acid	0.87	0.67	0.83	0.85	1.41	0.217	0.190	0.078	0.108
Proportions in total SCFA, %									
Butyric acid	10.05 ^b^	7.57 ^b^	8.32 ^b^	7.84 ^b^	13.24 ^a^	1.046	0.004	0.055	0.001
BCFA/SCFA	0.010 ^b^	0.050 ^a^	0.030 ^ab^	0.028 ^b^	0.013 ^b^	0.008	0.013	0.489	0.009

^1^ Total SCFA included acetate, propionate, and butyrate. ^2^ Total BCFA included isobutyrate and isovalerate. SCFA, short-chain fatty acid; BCFA, branch-chain fatty acid. ^a–c^ Values within a row with no common superscripts differ significantly (*p* < 0.05).

**Table 7:** The top 10 phyla in cecum digesta between groups (% of total sequences).

Phylum	Corn Bran Level, %		SEM	*p*-Value
	0	12		
Firmicutes	59.79	69.49	5.034	0.238
Bacteroidetes	19.91	13.19	3.424	0.190
Actinobacteriota	12.11	12.16	7.173	0.979
Deferribacteres	3.06 ^a^	0.32 ^b^	1.690	0.021
Unidentified Bacteria	2.62	2.32	0.447	0.639
Proteobacteria	0.22	0.72	0.214	0.473
Spirochaetota	0.50 ^a^	0.09 ^b^	0.158	0.045
Fusobacteriota	0.15 ^a^	<0.01 ^b^	0.082	0.037
Desulfobacterota	0.29	0.31	0.049	0.698
Cyanobacteria	0.06	0.04	0.029	0.628

^a,b^ Values within a row with no common superscripts differ significantly (*p* < 0.05).

**Table 8:** The top 10 genera in cecum digesta between groups (% of total sequences).

Genus	Corn Bran Level, %		SEM	*p*-Value
	0	12		
*Lactobacillus*	13.77	20.30	4.740	0.339
*Enterococcus*	7.70	2.73	3.675	0.399
*Bacteroides*	8.51	4.07	1.776	0.113
*Romboutsia*	3.32	5.55	2.314	0.535
*Pediococcus*	0.54 ^b^	7.42 ^a^	1.349	<0.001
*Subdoligranulum*	10.09	9.92	1.160	0.939
*Mucispirillum*	0.21	0.32	0.111	0.527
*Olsenella*	4.59	3.83	1.037	0.617
*Bifidobacterium*	4.04	2.20	0.698	0.082
*Rikenellaceae_RC9_gut_group*	3.36	2.15	0.477	0.085

^a,b^ Values within a row with no common superscripts differ significantly (*p* < 0.05).

## Data Availability

Data are contained within the article.
